# Supplementary material for: Analysis of SMAD1/5 target genes in a sea anemone reveals ZSWIM4-6 as a novel BMP signaling modulator
Source: eLife. 2024 Feb 7;13:e80803. doi: 10.7554/eLife.80803 (PMC10849676; doi:10.7554/eLife.80803)
Supplement: Supplementary file 3. — (A) ZSWIM4-6 guide RNA sequences and sequencing primers. (B) Primers used for cloning dominant-negative BMP receptor fragments. (C) Morpholino sequences. (D) Primer sequences for MO specificity testing. (E) Short hairpin RNA targets. (F) qPCR primers. (G) Primer sequences for in situ probes. [file elife-80803-supp3.docx]

**Supplementary File 3A - ZSWIM4-6 guide RNA sequences and sequencing primers**

| **Name** | **Sequence** |
| --- | --- |
| *zswim4-6_g254* | GCACACGTCCGTATGCCACG**CGG** |
| *zswim4-6_g282* | AAAAGACTGCCGTTTCAGAA**GGG** |
| zswim4-6_sqF | CTAGACCTAGCCGCGAAGTG |
| zswim4-6_sqR | TTTGCACCGACCGACTACAA |

**Supplementary File 3B - Primers used for cloning dominant-negative BMP receptor fragments**

| **Name** | **Sequence** |
| --- | --- |
| Alk2F | ATGCCGCCTTCTTTTGCTATAACCCTTGT |
| Alk2swF | TGCCCATGTAATGAGGAAAGAGGTGGTGGTGGTAGTGTGA |
| Alk2swR | TCACACTACCACCACCACCTCTTTCCTCATTACATGGGCA |
| Alk3/6F | ATGGCTTTAGCCACCAAATCAACCATTTTC |
| Alk3/6swF | CGCATTAACTTTATAAGCTCTGGTGGTGGTGGTAGTGTGA |
| Alk3/6swR | TCACACTACCACCACCACCAGAGCTTATAAAGTTAATGCG |
| bmpRIIF | ATGTCTTGGATTTGGAGATTCC |
| bmpRIswF | TTCCCTGGACTTGAGCCAGGGTGGTGGTGGTAGTGTGA |
| bmpRIswR | TCACACTACCACCACCACCCTGGCTCAAGTCCAGGGAA |

**Supplementary File 3C - Morpholino sequences**

| **Name** | **MO sequence** | **[C]** | **Reference** |
| --- | --- | --- | --- |
| BMP2-4MO (DPPMO) | GTAAGAAACAGCGTAAGAGAAGCAT | 300 μM | Saina et al., 2009; Genikhovich et al., 2015 |
| ChdMO | GATCCACTCACCATCTTTGCGAGAC | 300 μM | Saina et al., 2009; Genikhovich et al., 2015 |
| GDF5lMO | AGGTTATTTAGCCTGACCTTGATCG | 500 μM | Genikhovich et al., 2015 |
| GrmMO | CTCAACAGCTTCTTCAATGATCCGT | 500 μM | Genikhovich et al., 2015 |
| ZSWIM4-6MO | CCGTCCATAGCTTGTACTGATCGAC | 300 μM | this study |
| Control MO | CCTCTTACCTCAGTTACAATTTATA | 500 μM | Genikhovich et al., 2015 |

**Supplementary File 3D - Primer sequences for MO specificity testing**

| **Name** | **Sequence** |
| --- | --- |
| wtZSWIM4-6MOmCh_F | GTCGATCAGTACAAGCTATGGACGGTGTGAGCAAGGGCGAG |
| misZSWIM4-6MOmCh_F | GTGGATCTGTAGAACCTATGGATGGTGTGAGCAAGGGCGAG |

**Supplementary File 3E - Short hairpin RNA targets**

| **Name** | **Target sequence** |
| --- | --- |
| sh_alk2#1 | GTGGCAAGCCTAATCTACA |
| sh_alk2#2 | GGCATGACTTAGGACCAGT |
| sh_alk3/6#1 | GGCCAAGTCACTCAAATGT |
| sh_alk3/6#2 | GGAGAGGCGCATTAACTTT |
| sh_bmpRII#1 | GTCGAGAGGACCCTTACTT |
| sh_bmpRII#2 | GCTGGTACAGTCCAAGAAT |

**Supplementary File 3F - qPCR primers**

| **Name** | **forward** | **reverse** |
| --- | --- | --- |
| alk2q | TGTGTCGGAGGTGTGAACATC | TGTCTCTGTGCTGCAGGTTTG |
| alk3/6q | AGCGGAGGAAGAAGGCTATGA | TTCCTATGCACACTGGCAGG |
| bmpRIIq | TGGACGGGTCATGTGTCTTG | TAACGCAGTGTTCCCACCTC |
| Chd_genomic | tcaaaacaatagcaaagccaga | gcttgctgaccaaagacctc |
| Grm_genomic | ggcagccaatagctcttctg | gtcgatcgtcggaaagtagc |
| IntA_genomic | ttctgaatgaatgcggatga | ggatttctgctttgcgactc |

**Supplementary File 3G - Primer sequences for in situ probes**

| **NVE** | **Name** | **forward** | **reverse** |
| --- | --- | --- | --- |
| NVE25316 | *zswim4/6* | CGCATGCTCAGTCTGATCAC | CCAAGTCCTAGTCCGCTGAA |
| NVE15986 | *nocA* | AGAAATCACCGTTGGCCCTA | CTGCTAGACTGAACAGGGCT |
| NVE6154 | *pik* | GCATGCTGGACCTTTCTCAG | TCCATGACTGACGCTTTCCT |
| NVE20898 | *hmx3* | tcacaggcttaaaacgtcgc | acctgctatttgacCTACACCA |
| NVE14226 | *ephrin-b2* | AGCCCACAACACAATCAACA | aatcagcagggtgaaaatcg |
| NVE15989 | *nocA* | AGAAATCACCGTTGGCCCTA | CTGCTAGACTGAACAGGGCT |
| NVE22756 | *p63* | TGCCAACCACATCACAAGACA | GCTGATCACAGGGTTCCCAA |
| NVE18219 | *atoh7* | GCCCAGTCAAAACCACCAAC | TGTCATTCAGTGTTTGGTGACG |
| NVE16340 | *dusp1* | CCTACGCGGTGCAAACTTGT | AGGCGACTCTGCAGTTGGAA |
| NVE7831 | *morn* | AGTGGTTCTAGTGGAGCGCAA | CGTGCACGTCACTAAACAACG |
| NVE13995 | *mex3c* | GGGTCTTGTTGTCGGTCCTA | GCATACAGGGCAGACAGAGT |
| NVE6154 | *pik* | GCATGCTGGACCTTTCTCAG | TCCATGACTGACGCTTTCCT |
| NVE644 | *fnd3b* | AAGAGGAAGAAGGCCGAAGAGT | CTCCACTTACCGTGCTCTCATCT |
| NVE3152 | *tnf10* | CATGCTGCTAACTCGGTGTG | CCGCCCAAAGAATCGAAGTG |
| NVE5866 | *gsc* | TTTACGCCACAAAACGAGCG | ATTGACTCGCACATCTCGGG |
| NVE8464 | *hl1* | CCAAGCTGGAAAAAGCGGAC | GTTCTCTGCTTGGGAAGCCT |
| NVE22860 | *bmprII* | TGGACTTGAGCCAGCTAACA | TTGCTGACTTCTGGTTGTGC |
| NVE10444 | *isl-1* | GAAGACGTACTGCAAGCGAG | TATGACTCGCGGACTCAGAC |
